# Manifestation of the Purcell Effect in Current Transport through a Dot–Cavity–QED System

**DOI:** 10.3390/nano9071023

**Published:** 2019-07-17

**Authors:** Nzar Rauf Abdullah, Chi-Shung Tang, Andrei Manolescu, Vidar Gudmundsson

**Affiliations:** 1 Physics Department, College of Science, University of Sulaimani, Sulaimani 46001, Kurdistan Region, Iraq; 2 Komar Research Center, Komar University of Science and Technology, Sulaimani 46001, Kurdistan Region, Iraq; 3 Department of Mechanical Engineering, National United University, 2, Lienda, Miaoli 36063, Taiwan; 4 School of Science and Engineering, Reykjavik University, Menntavegur 1, IS-101 Reykjavik, Iceland; 5 Science Institute, University of Iceland, Dunhaga 3, IS-107 Reykjavik, Iceland

**Keywords:** quantum transport, quantum dot, cavity-quantum electrodynamics, quantum master equation, electro-optical effects

## Abstract

We study the transport properties of a wire-dot system coupled to a cavity and a photon reservoir. The system is considered to be microstructured from a two-dimensional electron gas in a GaAs heterostructure. The 3D photon cavity is active in the far-infrared or the terahertz regime. Tuning the photon energy, Rabi-resonant states emerge and in turn resonant current peaks are observed. We demonstrate the effects of the cavity–photon reservoir coupling, the mean photon number in the reservoir, the electron–photon coupling and the photon polarization on the intraband transitions occurring between the Rabi-resonant states, and on the corresponding resonant current peaks. The Rabi-splitting can be controlled by the photon polarization and the electron–photon coupling strength. In the selected range of the parameters, the electron–photon coupling and the cavity-environment coupling strengths, we observe the results of the Purcell effect enhancing the current peaks through the cavity by increasing the cavity–reservoir coupling, while they decrease with increasing electron–photon coupling. In addition, the resonant current peaks are also sensitive to the mean number of photons in the reservoir.

## 1. Introduction

Single photon sources have been widely sought after for research in fields of science and technology [1,2]. A single photon can be used to control optical quantum simulators [3,4] and multi-qubit gates [5], and it can also be coupled to an electronic structure such as a quantum dot (QD) to control electron motion [6]. In such systems, quantum mechanical methods are used to describe the light consisting of few photons, i.e., the light field has to be fully quantized [2,7]. A quantized photon system coupled to an electronic system can be used to explore many interesting aspect of physical problems and phenomena in the nanoscale range such as the Purcell effect [8,9,10,11], quantum information processing [12], quantum communication networks [13], quantum bit architectures based on simulate on-chip phonon-mediated interactions between strongly correlated electrons [14], photon-induced tunneling [15], plasmonic resonators [16] and resonance fluorescence [17].

Several parameters need to be considered when studying some types of interactions, such as electron–photon coupling strength, $g_{\gamma }$, [15], the coupling strength of the cavity–photon field to the environment, κ, [18,19] and mean value of photons in the environment with energy corresponding to the cavity mode, i.e., the temperature of the environment. The electron–photon coupling strength can be compared to the coupling strength of the cavity–photon field to the environment. If $g_{\gamma }>\kappa$, the system is called the strong coupling regime [20].

In the strong coupling regime, a QD system exposed to a quantized photon field has been found to be one of the most fascinating system for investigating several physical phenomena in modern nanodevices. The Rabi splitting and oscillations in a QD coupled to a photon field lead to observation current peaks which can be used to measure photoluminescence [21], vacuum effects, like the ground state electroluminescence [22,23], entanglement characteristics of a photon source with an electronic system [24], resonance fluorescence and Rayleigh scattering in Mollow-triplet-like spectra [25], and the photon-induced transport in a Rabi-splitting of a two level [26] and many level QD [27]. Furthermore, the strong coupling regime paves the way to industrial technology for building solid state-based quantum optical processors [28] and semiconductor chips [29,30].

An early interesting achievement in the field of quantum optics was the demonstration of the Purcell effect [31,32] which is the enhancement of a quantum system’s spontaneous emission rate by its environment. This phenomena has been investigated by many research group [33,34]. The emission intensity of spin-up exciton state with respect to spin-down exciton state is enhanced at resonance due to Purcell effect [9]. In addition, the enhancement of the Purcell effect has been achieved by controlling the effective coupling with the microcavity [35,36].

Motivated by the above mentioned scientific works, we modelled a two-dimensional electron system in a GaAs QD embedded in a short quantum wire coupled to two electron reservoirs [37]. The wire-dot system is also coupled to a 3D-cavity and the cavity is in turn coupled to a photon reservoir, i.e., the external environment. The electron transport in the steady-state is investigated under the effects of a cavity photon field using a Markovian general master equation (GME) [38]. Previously, we have investigated and reported the characteristics of electron transport in systems with different geometries using non-Markovian for the short time evolution, and Markovian master equations for the long time, including the coupling of electrons to a quantized photon field. We have studied Rabi-oscillations in the intermediate time regime [39], oscillations in the electron transport caused by Rabi-resonant states in the steady state [40], photon-assisted tunneling [41] and thermoelectric transport in the transient regime [42,43,44,45,46]. In addition, the photocurrent generated by photon replica states in an off-resonant dot-cavity system has been studied where the Rabi effect plays only a minor role in the transport [47]. The system is said to be in the off-resonant regime if the energy spacing between two specific lowest states of the QD system is smaller than the photon energy. It is also shown that the photocurrent can be manipulated by the photon polarization and the cavity–photon coupling strength to the environment. Furthermore, the resonant current peaks generated by Rabi-resonant states in a quantum dot have been investigated where only the influences of the photon polarization was highlighted. We have shown that the Rabi effect has a major impact on the transport [40]. In the current work, we present a general picture of the influences of the electron–photon coupling strength, the cavity–reservoir coupling strength and the mean photon number in the photon reservoir on the transport properties of a QD system in the steady-state regime in which the Rabi-effect has a large role. We assume a strong coupling regime, ($g_{\gamma }>\kappa$), and investigate the resonant current generated by the multiple resonance states. An enhancement of the current through the QD system is observed, which is demonstrated to be a direct consequence of the Purcell effect.

## 2. Hamiltonian of the Total System

We assume a QD embedded in a two dimensional short quantum wire in the xy-plane with hard walls at the ends in the *x*-direction and the parabolic confinement potential in the *y*-direction. The wire-QD system is exposed to a constant external weak magnetic field and coupled to a photon cavity with a single photon mode. In order to pump electrons to and from the QD system, we assume the QD system coupled to two electron reservoirs via a tunneling region called the coupling or contact region [48,49]. The Hamiltonian of the total system, the QD system and the cavity, in the many-body basis can be defined as

(1) $${\hat{H}}_{S}={\hat{H}}_{e}+{\hat{H}}_{\gamma }+{\hat{H}}_{e-\gamma },$$

where ${\hat{H}}_{e}$ is the Hamiltonian of the QD system, ${\hat{H}}_{\gamma }$ indicates the Hamiltonian of the free photon field, and ${\hat{H}}_{e-\gamma }$ defines the interaction between the QD system and the cavity. We start with the Hamiltonian of the QD system which is given by

(2) $$\begin{array}{ccc} {\hat{H}}_{e} & = & \sum_{nn^{\prime }}^{}\langle \Psi_{n^{\prime }}|\left[\frac{{\hat{\pi }}_{e}}{2m_{\mathrm{eff}}}+eV_{g}+V_{\mathrm{QD}}\right]|\Psi_{n}\rangle d_{n^{\prime }}^{†}d_{n}\\ & + & H_{Z}+\frac{1}{2}\sum_{\begin{array}{c} nn^{\prime }\\ mm^{\prime } \end{array}}V_{nn^{\prime }mm^{\prime }}\;d_{n^{\prime }}^{†}d_{m^{\prime }}^{†}d_{m}d_{n}. \end{array}$$

Herein, $\Psi_{n}$ stands for a single electron eigenstate, $m_{\mathrm{eff}}$ is the effective mass of electrons, and ${\hat{\pi }}_{e}=:p+\frac{e}{c}A_{B}$, where p is the canonical momentum operator, $A_{B}=-By\hat{x}$ is the magnetic vector potential with $B=B\hat{z}$. $m_{\mathrm{eff}}=0.067\;m_{e}$ is the effective mass of an electron in GaAs-based material with relative dielectric constant $\epsilon_{r}=12.4$. $V_{g}$ is the gate voltage that shifts the energy states of the QD system with respect to the chemical potentials of the leads, $d^{†}\left(d\right)$ are the fermionic creation (annihilation) operators, and $V_{\mathrm{QD}}$ is the potential that forms the quantum dot given by

(3) $$V_{\mathrm{QD}}=V_{0}\;e^{\left(-\gamma_{x}^{2}x^{2}-\gamma_{y}^{2}y^{2}\right)},$$

where $V_{0}$ is the depth of the QD, $\gamma_{x}$ and $\gamma_{y}$ together with $V_{0}$ determine the diameter of the QD. We assume $V_{0}=-3.3$ meV and $\gamma_{x}=\gamma_{y}=0.03$ nm${}^{-1}$ in our calculations.

The first term of the second line of Equation (2) is the Zeeman Hamiltonian, $H_{Z}=g^{*}\mu_{B}B\sigma_{z}/2$, and the second term represents the Coulomb interaction, while $V_{nn^{\prime }mm^{\prime }}$ are the Coulomb integrals

(4) $$V_{nn^{\prime }mm^{\prime }}=\langle \Psi_{n^{\prime }}\Psi_{m^{\prime }}|\frac{e^{2}}{\bar{\kappa }\left|r-r^{\prime }\right|}|\Psi_{n}\Psi_{m}\rangle ,$$

where $\bar{\kappa }$ is the dielectric constant, and $|r-r^{\prime }|$ the spatial separation of an electron pair. An exact diagonalization technique in a truncated Fock space is used to obtain the many-electron states.

The second term of Equation (1) defines the free photon field

(5) $${\hat{H}}_{\gamma }=\hbar \omega_{\gamma }\;a^{†}a$$

with $\hbar \omega_{\gamma }$ being the single photon energy and $a^{†}$ and *a* the bosonic creation and annihilation operators, respectively. The last term of Equation (1) is

(6) $$\begin{array}{cc} {\hat{H}}_{e-\gamma } & =\frac{e}{m_{\mathrm{eff}}}\sum_{nn^{\prime }}\langle \Psi_{n^{\prime }}|\pi_{e}\cdot A_{\gamma }|\Psi_{n}\rangle \;d_{n^{\prime }}^{†}d_{n}\\ & +\frac{e^{2}{A_{\gamma }}^{2}}{2m_{\mathrm{eff}}}\sum_{nn^{\prime }}\langle \Psi_{n^{\prime }}|\Psi_{n}\rangle \;d_{n^{\prime }}^{†}d_{n}, \end{array}$$

which is the Hamiltonian of the electron–photon interactions including both the paramagnetic Hamiltonian ($A_{\gamma }$ term) and the diamagnetic Hamiltonian ($A_{\gamma }^{2}$ term). The photon field interacting with the QD system is represented via the vector potential

(7) $$A_{\gamma }=A\left(e\;a+e^{*}\;a^{†}\right)$$

with $e=e_{x}$ for *x*-polarized photon field and $e=e_{y}$ for *y*-polarized photon field. The amplitude of the photon field, *A*, is related to the electron–photon coupling strength via $g_{\gamma }=eAa_{w}\Omega_{w}/c$, where $a_{w}$ is the effective magnetic length, *e* displays the electron charge, and $\Omega_{w}$ refers the effective confinement frequency of electrons in the QD system. We consider the wavelength of the photon field in the cavity to be much larger than the size of the electronic system composed of the short wire and the QD. A numerically exact diagonalization procedure is used for the electron–photon interaction using the Coulomb interacting many-body bases obtained earlier [48].

The QD system is coupled to two leads from the left and right sides, with different chemical potential. Therefore, electrons can flow from the leads to the QD system, and vice versa. To calculate the electron motion through the system in the steady-state regime, a Markovian quantum master equation is utilized. The derivation of the master equation formalism starts with the projection formalism of Nakajima and Zwanzing [50,51]. The resulting non-Markovian generalized master equation with an integral kernel evaluated up to second order in the system-lead coupling delivers the reduced density operator for the central system. As we are interested in the steady-state we apply a Markovian approximation to the GME and transform it to Liouville space of transitions [38]. One assumes the initial reduced density operator of the QD system to be ${\hat{\rho }}_{S}\left(t_{0}\right)$ and for the leads it is ${\hat{\rho }}_{l}\left(t_{0}\right)$. Before the coupling between the QD system and the leads, the total density operator is assumed to be a tensor product of the uncorrelated sub parts $\hat{\rho }\left(t_{0}\right)={\hat{\rho }}_{l}\left(t_{0}\right){\hat{\rho }}_{S}\left(t_{0}\right)$. After the coupling, for $t>t_{0}$, we can write the reduced density operator of the QD system as ${\hat{\rho }}_{S}\left(t\right)={\mathrm{Tr}}_{l}\left(\hat{\rho }\right)$ with *l* indicating the left (L), the right (R) leads and the photon reservoir. For the electron–photon interaction in the QD system we do not use the rotating wave approximation, but we do so for the photon–cavity environment coupling. In order to do that properly we have taken care to rid the annihilation (creation) operator for the cavity photons in the dissipation terms of the master equation of all high frequency creation(annihilation) terms when casting the master equation into the fully interacting basis of cavity–photon dressed many-electron states [52,53,54].

Once we get the reduced density operator of the QD system, the current going through it can be calculated using

(8) $$I_{L,R}=:\mathrm{Tr}\left[{\hat{\dot{\rho }}}_{S}^{L,R}\left(t\right)\hat{Q}\right].$$

where $Q=-e\sum_{i}d_{i}^{†}d_{i}$ is the charge operator of the QD system with ${\hat{d}}^{†}\left(\hat{d}\right)$ the electron creation (annihilation) operator of the QD system, respectively [55].

## 3. Results

**Figure 1:**
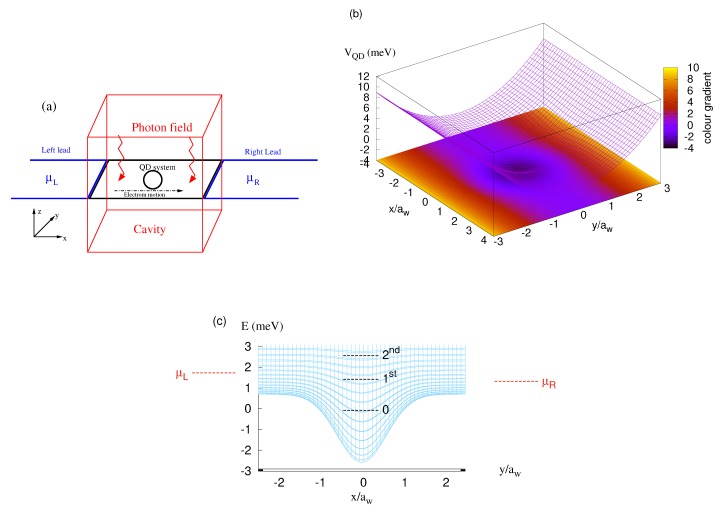
(**a**) Schematic diagram demonstrates the QD system (black) connected to the leads (blue) where the chemical potential of the left lead ($\mu_{L}$) is higher than the that of the right lead ($\mu_{R}$). The red zigzags indicate the quantized photon field in the cavity (red rectangle). (**b**) The potential $V_{\mathrm{QD}}$ defining the QD embedded in a short quantum wire that is connected diametrically to the leads in the *x*-direction. (**c**) The potential $V_{\mathrm{QD}}$ in relation to the chemical potentials of the leads and the three lowest one-electron states of the system.

The main results of our calculations are presented in this section. We consider the diameter of the quantum dot to be d=66.5 nm and the length of the quantum wire to be $L_{x}=150$ nm. A weak external magnetic field is applied to the total system, the QD system and the leads, B=0.1 T, which is perpendicular to the two-dimensional plane of the electron motion. The magnetic field is weak enough to avoid most of the influences of Lorentz force on the orbital motion and lifts the spin degeneracy of the system. The chemical potentials are assumed to be $\mu_{L}=1.65$ meV and $\mu_{R}=1.55$ meV here, and the temperature of the leads is fixed at $T_{L,R}=0.5$ K. The schematic diagram of the QD system (black) coupled to the leads (blue) and the cavity (red) is shown in Figure 1a where the red zigzags indicate the quantized photon field, and the potential of QD embedded in a quantum wire is presented in Figure 1b. In addition, the potential $V_{\mathrm{QD}}$ in relation to the chemical potentials of the leads and the three lowest one-electron states of the system is demonstrated in Figure 1c.

We intend to show the influence of tuning the photon energy, $\hbar \omega_{\gamma }$, the photon polarization, the cavity–reservoir coupling strength, κ, and the mean photon number in the reservoir, $n_{R}$, on the transport properties of the QD system. The two physical parameters, κ and $n_{R}$, are included in the Markovian master equation which is not presented here [49].

**Figure 2:**
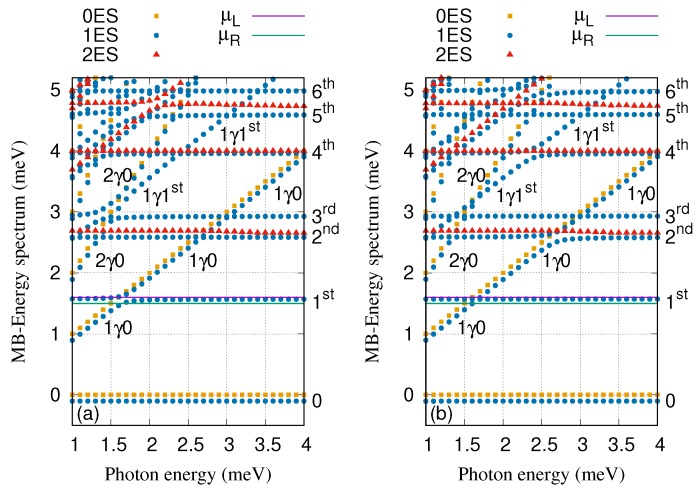
Many-Body energy spectra of the cavity-QD system versus the photon energy for *x*- (**a**) and *y*-polarized (**b**) photon field, where brown squares refer to zero-electron states (0ES), blue circles display one-electron states (1ES), and red triangles are two-electron states (2ES). The chemical potential of the left and the right leads are $\mu_{L}=1.65$ meV (purple line) and $\mu_{R}=1.55$ meV (green line), respectively. 0 is the one-electron ground-state energy, 1γ0 and 2γ0 demonstrates the one- and two-photon replica of the 0, and 1st, 2nd, 3rd, 4th, 5th, 6th indicate the one-electron first-, second-, third-, fourth-, fifth- and sixth-excited state, respectively. The 1γ1st indicates the one-photon replica state of the 1st. The photon number initially in the reservoir $n_{R}=1$, $g_{\gamma }=0.1$ meV, and $\kappa =10^{-5}$ meV. The magnetic field is B=0.1T, $eV_{g}=0.651$ meV, $T_{L,R}=0.5$ K and $\hbar \Omega_{0}=2.0\;\mathrm{meV}$.

The energy spectrum of the QD system coupled to the cavity in the *x*- (a) and *y*-polarized (b) photon field is displayed in Figure 2, where the electron–photon coupling strength is $g_{\gamma }=0.1$ meV, the cavity–reservoir coupling strength is $\kappa =10^{-5}$ meV and the mean photon number in the reservoir is $n_{R}=1$. The six lowest states of the QD system are found in the selected range of the energy between −1 meV and 5.2 meV. The states of the system are classified as follows: The zero-electron states, 0ES (brown squares), the one-electron states, 1ES (blue circles), and the two-electron states, 2ES (red triangles). In addition, the labels appearing in the figures, 0, 1γ0, and 2γ0 display the ground-state, the first and second photon replica of the ground-state, respectively, while 1st and 1γ1st are the first-excited state and the first photon replica of first-excited states, respectively. The photon dressed many-electron states are photon replica states which have a mean number of photons close to integers if they are not states in a Rabi-split pair. The rest of labels such as 2nd, 3rd, 4th, 5th, and 6th indicate the second-, third-, fourth-, fifth-, and sixth-excited states, respectively. We found that each electronic state in the energy spectrum contains a spin components that are Zeeman split due to the external field B=0.1 T. The chemical potentials of the leads (purple and green lines) are arranged in the way that the first-excited state, 1st, is located in the bias window.

By changing the photon energy, anti-crossings between the energy states are formed especially at photon energies 1.5, 1.7, 2.7, 3.0, and 3.4 meV. The photon-exchange between the two states forming the anti-crossings confirms Rabi-splittings [40]. It is clearly seen that the Rabi-splittings is influenced by the photon polarization. Therefore, the energy splitting between 1γ0 and 1st at the photon energy 1.7 meV for the *x*-polarization is larger than that of the *y*-polarization. The reason is that the wavefuntion of 1st is localized along the *x*-direction around the QD and the *x*-polarized photon field will thus be more effective in polarizing the charge [42]. Contrary, the energy splitting between 1γ0 and 2nd at the photon energy 2.7 meV for the *y*-polarization is larger than that of the *x*-polarization because 2nd is more delocalized to the *y*-direction. This indicates that the geometry of the states plays an important role as some states are more polarizable in the *x*-direction and some other states in the *y*-direction.

**Figure 3:**
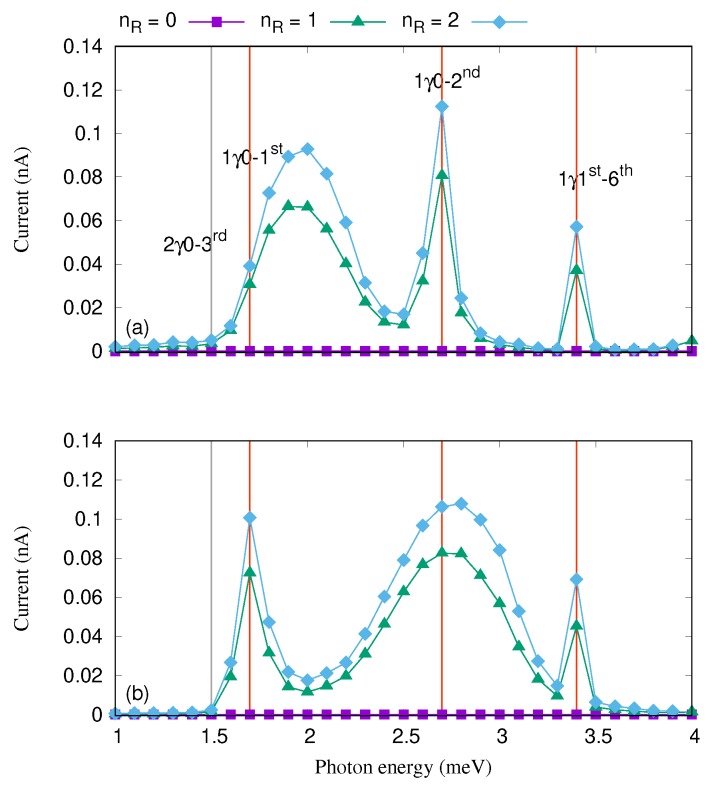
Current versus the photon energy is plotted for the photon number $n_{R}=0$ (purple squares), 1 (green triangles), and 2 (blue diamonds) in the case of *x*- (**a**) and *y*-polarized (**b**) photon field with $g_{\gamma }=0.1$ meV, and $\kappa =10^{-5}$ meV. The vertical red lines indicates the positions of the resonance states. The chemical potential of the left lead is $\mu_{L}=1.65$ meV and the right lead is $\mu_{R}=1.55$ meV. The external magnetic field is B=0.1T, $eV_{g}=0.651$ meV, $T_{L,R}=0.5$ K, and $\hbar \Omega_{0}=2.0\;\mathrm{meV}$.

The influence of photon polarization on the current in the QD system was reported in [40], where several peaks in the current were observed by changing the photon energy. Similar idea is also used in this study. Tuning the photon energy several peaks in the current are found indicating resonances as it is shown in Figure 3 in the case of $n_{R}\neq 0$. The main peaks are found at the photon energy 1.7 meV representing a transition between 1γ0 and 1st, at 2.7 meV for transition between 1γ0 and 2nd, and at 3.4 meV for transition between 1γ1st and 6th corresponding to the Rabi-splittings shown in Figure 2. We observed that the broadening of the current peaks depends on the strength of the corresponding Rabi-splitting [56]. In the weak coupling regime for the central system and the leads the broadening depends to largest extent on the strength of the corresponding Rabi-splitting. The coupling to the leads is state dependent, it depends on the geometry of the corresponding electron states in the central system and the leads. This coupling could thus also contribute to the broadening, but as we take care to stay well in the weak coupling limit for the states active in the transport the broadening can be attributed mostly to the strength of the Rabi splitting. Therefore, the broadening of the current peak corresponding to the resonance between 1γ0 and 1st at the photon energy 1.7 meV for the *x*-polarization is larger than that of the *y*-polarization. This is caused by the Rabi-splitting between 1γ0 and 1st for the *x*-polarization is larger than that of the *y*-polarization (see Figure 2). In contrast to the mentioned current peak, the broadening of the current peak formed at the photon energy 2.7 meV is larger for the *y*-polarization. The reason is that the Rabi-splitting between 1γ0 and 2nd is larger for the *y*-polarization than the *x*-polarization. As we have mentioned before, the geometry of the states plays an essential role here. The first-excited state is more polarizable in the *x*-direction while the second-excited state by contrast is more polarizable in the *y*-direction. We should mention that an intraband transition occurs between the aforementioned resonant states and it has a major role in the current transport [40]. These intraband transitions can be tuned by other physical parameter of the system such as the electron–photon coupling strength and the cavity–photon reservoir coupling strength.

Tuning the photon number in the photon reservoir, $n_{R}$, and see it’s influence on the current transport properties of the QD system in Figure 3, where the cavity–reservoir coupling strength is assumed to be $10^{-5}$ meV and $g_{\gamma }=0.1$ meV.

As the photon number is increased the participation of the photon replicas in the electron transport is enhanced. As a result, the current is slightly increased for the case of two photons (green diamonds) for both photon polarizations. This happens as the photon replicas are not pure simple perturbational states with an integer number of photons, but instead contain states with 0, 1, and 2 photons at least to some amount.

It should be noted that if the mean number of photon is zero, $n_{R}=0$, the current is very close to zero which is due to inactivated photon replica states in the transport in the absence of flow of photons into the cavity from the reservoir. In addition, the QD system is in a Coulomb blocking regime in the steady state when $n_{R}=0$ and the charging of 1γ0 and 1γ1st is thus approaching zero. These effects lead to a vanishing current.

**Figure 4:**
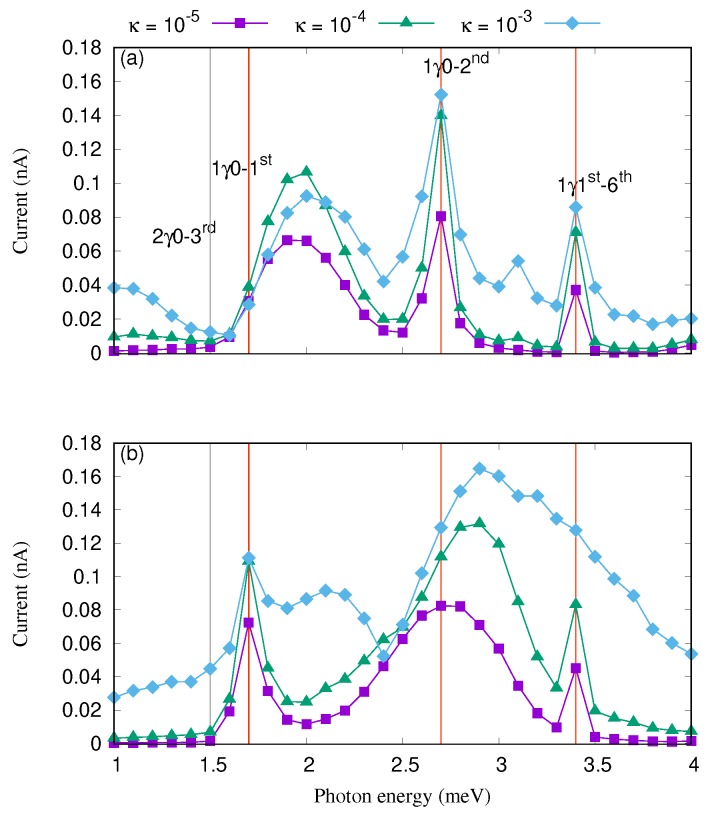
Current versus the photon energy for the cavity–reservoir coupling $\kappa =10^{-5}$ meV (purple squares), $10^{-4}$ (green triangles), and $10^{-3}$ (blue diamonds) in the case of *x*- (**a**) and *y*-polarized (**b**) photon field with $g_{\gamma }=0.1$ meV, and $n_{R}=1$. The vertical red lines indicates the positions of the resonance states. The chemical potential of the leads are $\mu_{L}=1.65$ meV, and $\mu_{R}=1.55$ meV. The external weak magnetic field is B=0.1T, $eV_{g}=0.651$ meV, $T_{L,R}=0.5$ K, and $\hbar \Omega_{0}=2.0\;\mathrm{meV}$.

We further investigate the transport characteristics by tuning the cavity–reservoir coupling and fix the photon number in the reservoir. Figure 4 shows the current versus the photon energy for different values of the cavity–reservoir coupling strength in the case of *x*- (a) and *y*-polarized (b) photon field where the electron–photon coupling strength is fixed at $g_{\gamma }=0.1$ meV and the mean value of photons with the particular energy in the reservoir is $n_{R}=1$, respectively.

The current is enhanced with the cavity–reservoir coupling overall for both photon polarizations. This shows that the cavity–reservoir coupling influences the intraband transitions that occur between the resonance states forming the Rabi-resonant pairs.

**Figure 5:**
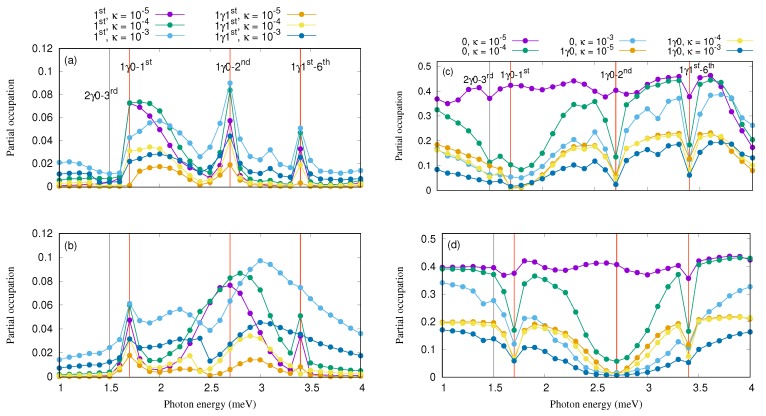
The partial occupation of the spin-up component of 1st and 1γ1st for different cavity–reservoir coupling is plotted *x*- (**a**) and *y*-polarized photon field (**b**). Furthermore, the partial occupation of the spin-up component of 0 and 1γ0 for different cavity–reservoir coupling is presented for *x*- (**c**) and *y*-polarized photon field (**d**). The cavity–reservoir coupling is assumed to be $\kappa =10^{-5}$ (purple for 0, and brown for 1st), $10^{-4}$ (green for 0, and yellow for 1st), and $10^{-3}$ (light blue for 0, and dark blue for 1st) in the case of *x*- (**a**) and *y*-polarized (**b**) photon field with $g_{\gamma }=0.1$ meV, and $n_{R}=1$. The vertical red lines indicates the positions of the resonance states. The chemical potential of the leads are $\mu_{L}=1.65$ meV and $\mu_{R}=1.55$ meV. The magnetic field is B=0.1T, $eV_{g}=0.651$ meV, $T_{L,R}=0.5$ K, and $\hbar \Omega_{0}=2.0\;\mathrm{meV}$.

In order to explain the current enhancement, we refer to the partial occupation of the most active states in the transport which are the first-excited state, 1st, and the first excitation thereof, 1γ1st, in Figure 5 for the *x*- (a) and *y*-polarization (b) on one hand, and on the other hand, the occupation of the ground-state, 0, and the first-excitation thereof, 1γ0, is presented in Figure 5 for the *x*- (c) and *y*-polarized photon field (d). We should mention that the Figure 5 shows only the spin-up component of the corresponding states, and the spin-down component is qualitatively the same (not shown).

Increasing the coupling strength of cavity–photon reservoir, the occupation of the first-excited state and the first excitation thereof is enhanced for both direction of the photon polarization while the occupation of the ground state and the excitation thereof is suppressed especially for the Rabi-resonant states. The first indication of charging of 1st and 1γ1st, and discharging of 0 and 1γ0 for the Rabi-resonant states is a confirmation of the intraband transition occurring between the states.

Increasing the cavity–photon reservoir coupling strength, these intraband transitions become weak especially at $\kappa =10^{-3}$. Therefore, the current going through 0 and 1γ0 is almost blocked but the current via 1st, and 1γ1st is increased which in turn increase the total current through the QD system because 1st is confined in the bias window. We are seeing here a manifestation of the Purcell effect, that was originally stated about the enhancement of radio wave emission of atoms in photon–cavities [57], but here it manifests itself in the enhanced current peaks of electrons through the cavity.

The last test of our calculation is the influences of electron–photon coupling strength between the electrons in the quantum dot system and the photons in the cavity on the transport properties.

**Figure 6:**
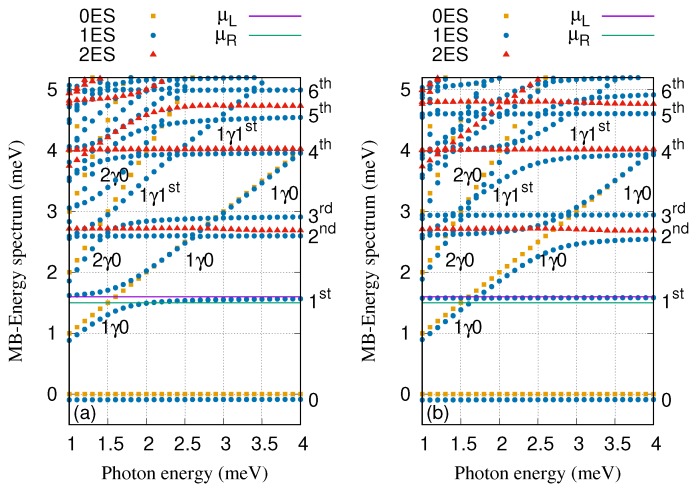
Many-Body energy spectra of the cavity-QD system versus the photon energy for *x*- (**a**) and *y*-polarized (**b**) photon field, where brown squares refer to zero-electron states (0ES), blue circles display one-electron states (1ES), and red triangles are two-electron states (2ES). The chemical potential of the left and the right leads are $\mu_{L}=1.65$ meV (purple line) and $\mu_{R}=1.55$ meV (green line), respectively. 0 is the one-electron ground-state energy, 1γ0 and 2γ0 demonstrates the one- and two-photon replica of the 0, and 1st, 2nd, 3rd, 4th, 5th, 6th indicate the one-electron first-, second-, third-, fourth-, fifth- and sixth-excited state, respectively. The 1γ1st indicates the one-photon replica state of the 1st. The photon number initially in the reservoir $n_{R}=1$, $g_{\gamma }=0.3$ meV, and $\kappa =10^{-5}$ meV. The magnetic field is B=0.1T, $eV_{g}=0.651$ meV, $T_{L,R}=0.5$ K and $\hbar \Omega_{0}=2.0\;\mathrm{meV}$.

Figure 6 displays the many-body energy spectrum of the QD system coupled to the cavity for both *x*- (a) and *y*-polarized (b) cavity–photon field where the electron–photon coupling strength is tuned to $g_{\gamma }=0.3$ meV. Comparing to the energy spectrum presented in Figure 2, where the electron–photon coupling strength is weaker, $g_{\gamma }=0.1$ meV, some changes in the energy spectrum can be seen [47,58]. For instance, the Rabi-splitting between 1st and 1γ0 at the photon energy 1.7 meV becomes larger here for the *x*-polarized photon field (see Figure 6a). Furthermore, the Rabi-splitting between 1γ0 and 2nd at the photon energy 2.7 meV here is much larger for the *y*-polarization comparing to the case when $g_{\gamma }=0.1$ meV (see Figure 6b). The first-photon replica state, 1γ1st, is not resonant with the sixth-excited state, 6th, anymore here while a strong Rabi-splitting between these two state was seen at $g_{\gamma }=0.1$ meV (see Figure 2) especially for the *y*-polarization.

**Figure 7:**
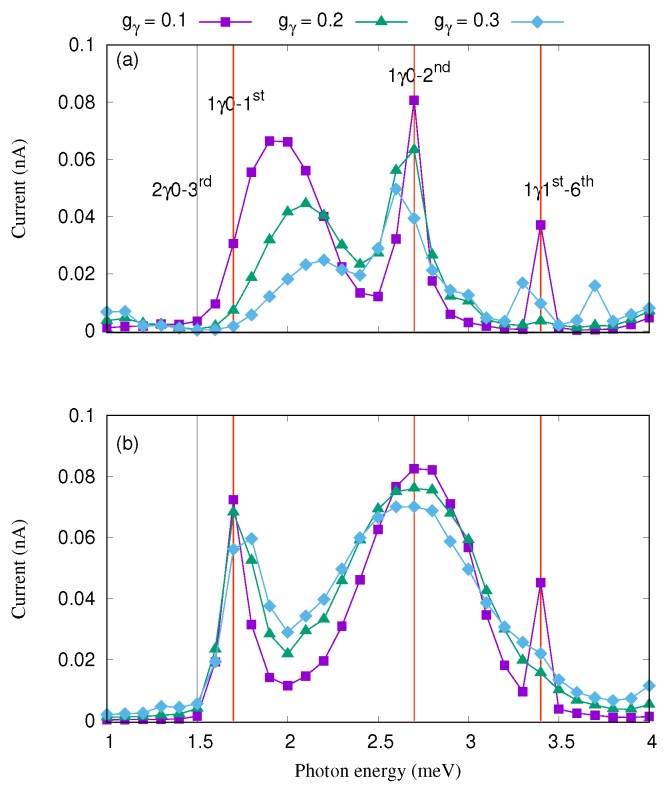
Current versus the photon energy for $g_{\gamma }=0.1$ (purple squares), 0.2 (green triangles), and 0.3 meV (blue diamonds) in the case of *x*- (**a**) and *y*-polarized (**b**) photon field. The cavity–reservoir coupling is $\kappa =10^{-5}$, and $n_{R}=1$. The vertical red lines indicates the positions of the main resonance states. The chemical potential of the left leads are $\mu_{L}=1.65$ meV and $\mu_{R}=1.55$ meV. The magnetic field is B=0.1T, $eV_{g}=0.651$ meV, $T_{L,R}=0.5$ K, and $\hbar \Omega_{0}=2.0\;\mathrm{meV}$.

The current as a function of the photon energy for three values of the electron–photon coupling strength is shown in Figure 7 for the *x*- (a) and *y*-polarized (b) photon field.

The current decreases with increasing electron–photon coupling strength for both direction of photon polarization. We start with the case of *x*-polarization (see Figure 7a), the current suppression in the leftmost peak is observed at high $g_{\gamma }=0.3$ meV (blue squares) which is due to the larger Rabi-splitting between 1st and 1γ0 for the photon energy 1.7 meV. The Rabi oscillation between these two states is thus increased and in turn the current is diminished. Furthermore, the positions of the two other peaks are shifted at $g_{\gamma }=0.3$ meV since the locations of Rabi-splitting forming the two peaks are moved.

For the *y*-polarized photon field (Figure 7b), the current of the leftmost peak is slightly changed with electron–photon coupling strength because the Rabi-splitting of the corresponding states is not much influenced by the photon polarization as is shown in Figure 2b and Figure 6b. In addition, the broadening of the current peak formed due to the Rabi-splitting between 1γ0 and 2nd at 2.7 meV is increased at higher electron–photon coupling strength. The last current peak at the photon energy 3.4 meV vanishes since the anti-crossing between 1γ1st and 6^th^ is not found anymore at $g_{\gamma }=0.3$ meV (see Figure 6b).

## 4. Summary

To summarize our results, we have shown that the photon polarization, the electron–photon coupling strength, the coupling strength of cavity–photon reservoir, and the mean photon number in the environment/reservoir can be used to control the resonance current peaks emerging due to the Rabi-resonant states of a quantum dot system coupled to a photon cavity and an external photon reservoir. We show that the photon polarization and the electron–photon coupling strength play an important role in the forming of Rabi-resonant states which in turn generate resonant current peaks. Furthermore, increasing the cavity photon coupling to the environment, κ, opening for faster flow of photons into and out of the cavity, the photon replica states are further activated leading to enhancement the electron transport. This phenomena demonstrates the Purcell effect [57] observed through current transport. Finally, by tuning the cavity–photon coupling strength the intraband transition between the Rabi-resonant states can be controlled.

## Abbreviations

The following abbreviations are used in this manuscript:

QD	Quantum Dot
$T_{L}$	Temperature of the left lead
$T_{R}$	Temperature of the right lead
MB	Many-Body states
0	Ground-state energy
1γ0	one-photon replica of the ground-state
2γ0	two-photon replica of the ground-state
1st	first-excited state
1γ1st	one-photon replica of the first-excited state
2nd	second-excited state
3rd	third-excited state
4th	fourth-excited state
5th	fifth-excited state
6th	sixth-excited state
